# Effect of PDCA-optimized good limb positioning on hemiparetic rehabilitation outcomes in acute cerebral infarction

**DOI:** 10.3389/fneur.2025.1554384

**Published:** 2025-06-06

**Authors:** Wei Liu, Guangyan Yao, Zhihui Song, Xin He

**Affiliations:** Department of Neurology, Central Hospital Affiliated to Shandong First Medical University, Jinan, China

**Keywords:** acute cerebral infarction, good limb positioning, PDCA cycle, early rehabilitation, functional recovery

## Abstract

**Background:**

Proper limb positioning plays a vital role in the early rehabilitation of patients with acute cerebral infarction (ACI), preventing complications such as muscle atrophy and joint contractures while promoting functional recovery. However, inconsistent implementation limits its effectiveness. This study evaluates the impact of the Plan-Do-Check-Act (PDCA) cycle management model in optimizing good limb positioning and improving rehabilitation outcomes.

**Methods:**

A prospective cohort study was conducted involving 300 hemiplegic ACI patients, with 150 patients receiving standard limb positioning care (control group) and 150 patients treated using the PDCA-optimized protocol (intervention group). The study was approved by The Ethics Committee of Central Hospital Affiliated to Shandong First Medical University (approval number: 20241104006). Outcomes included adherence rates, self-efficacy, quality of life (SF-36), activities of daily living (ADL), and secondary complications such as limb spasticity.

**Results:**

The intervention group demonstrated significantly higher adherence rates (88.0% vs. 48.0%, *p* < 0.001) and improved rehabilitation outcomes, including increased self-efficacy (25.0 vs. 17.0, *p* < 0.001), better quality of life (66.5 ± 13.8 vs. 61.7 ± 17.2, *p* < 0.001), and enhanced ADL scores (62.2 ± 10.2 vs. 52.8 ± 9.9, *p* < 0.01). Median hospital stay was reduced (10 days vs. 12 days, *p* = 0.001), and limb spasticity incidence was lower in the intervention group (*p* = 0.001). No significant differences in discharge NIHSS scores were observed.

**Conclusion:**

The PDCA cycle significantly enhances the implementation of good limb positioning, improving functional recovery, reducing secondary complications, and optimizing rehabilitation timelines for ACI patients. This study highlights the utility of PDCA in standardizing care practices and promoting better clinical outcomes. Further research should explore its broader application in diverse clinical settings.

## Introduction

Acute Cerebral Infarction (ACI), a prevalent condition among the middle-aged and elderly populations, is associated with high mortality, recurrence, and disability rates (1). Of those who survive, more than half experience varying degrees of functional impairment. The most common functional deficits include visual field defects, sensory and motor dysfunctions, speech and swallowing difficulties, as well as cognitive and psychological impairments (2). Furthermore, ACI survivors often face shoulder problems and urinary or bowel dysfunction. The rehabilitation process for ACI primarily targets restoring motor, cognitive, speech, and functional abilities, with an emphasis on improving the patient’s overall quality of life.

Among the various rehabilitation strategies, the importance of good limb positioning has garnered significant recognition in recent years. Clinical evidence supports that appropriate limb positioning not only aids in the prevention of complications such as muscle atrophy, shoulder-hand syndrome, and joint contractures, but also contributes to reducing the severity of limb spasticity after stroke, with studies showing a pooled prevalence of spasticity at 25.3% and indicating that proper limb positioning can significantly lower spasticity levels (3–5). Early rehabilitation, especially within the first month after ACI onset and thrombolysis, is crucial, as the brain exhibits higher plasticity during this period (6). For hospitalized ACI patients, early intervention plays a key role in enhancing motor function in the affected limb, reducing common secondary complications, and promoting favorable conditions for subsequent rehabilitation during the recovery phase (7, 8). However, despite the established benefits, the application of good limb positioning remains inconsistent in clinical practice, often hindered by a range of systemic, clinical, and environmental barriers. Many ACI patients do not receive optimal early rehabilitation, which can significantly impede functional recovery and the prevention of secondary complications.

The Plan-Do-Check-Act (PDCA) cycle, a widely recognized model for continuous quality improvement (CQI), has proven effective in addressing such challenges in various healthcare settings (9). Initially developed for business management, the PDCA framework has since been adapted to healthcare quality management, offering a structured approach to improving clinical practices and patient outcomes. By systematically identifying problems, implementing targeted interventions, monitoring progress, and refining strategies, the PDCA cycle has become a cornerstone in hospital management, achieving substantial improvements in patient care (10, 11). In the context of ACI rehabilitation, the PDCA framework can be particularly beneficial in optimizing the application of good limb positioning. In our institution, we have identified several obstacles that hinder the effective implementation of this rehabilitation technique, including insufficient multidisciplinary collaboration, inadequate training, and a lack of standardization in the approach. As a result, early rehabilitation therapy remains underutilized, particularly for ACI patients in the neurology department.

This study is the first to explore the application of the PDCA management model in optimizing good limb positioning for early functional recovery in hemiplegic patients following acute cerebral infarction ACI. The findings of this study may provide valuable insights into how quality improvement initiatives can be effectively integrated into rehabilitation protocols for ACI patients, offering a pathway to better recovery and long-term functional independence.

## Methodology

### Study design

A prospective cohort study was conducted in compliance with the principles of the Declaration of Helsinki and was approved by the institutional ethics committee of Central Hospital Affiliated to Shandong First Medical University (approval number: 20241104006). Written informed consent was obtained from all participants or their legal guardians, who were free to withdraw from the study without affecting their access to further treatment.

The study involved hospitalized patients with hemiplegia following thrombolysis for ACI in the neurology department. Patients admitted between January 1, 2022, and June 30, 2022, were designated as the control group (*n* = 150), while those admitted between July 1, 2022, and December 31, 2022, were assigned to the study group (*n* = 150).

#### Inclusion and exclusion criteria

Patients were included if they met the diagnostic criteria for acute stroke confirmed by CT or MRI, received thrombolysis therapy, and exhibited hemiparesis with an NIHSS score of at least 4 after thrombolysis. In addition, patients had to be conscious, have no significant cognitive impairment, be able to communicate effectively, and be aged between 30 and 80 years. Exclusion criteria included severe complications or comorbidities contraindicating early rehabilitation through good limb positioning, clinical deterioration or death preventing participation, and refusal to engage in early rehabilitation.

### Good limb positioning in the control group

From January to June 2022, standard good limb positioning was provided to the control group, in accordance with the Guidelines for Adult Stroke Rehabilitation and Recovery from the American Heart Association/American Stroke Association (12). Post-thrombolysis, patients underwent rehabilitation education and training under the guidance of ward medical staff (neurologists, rehabilitation physicians, and nurses).

**Figure 1:**
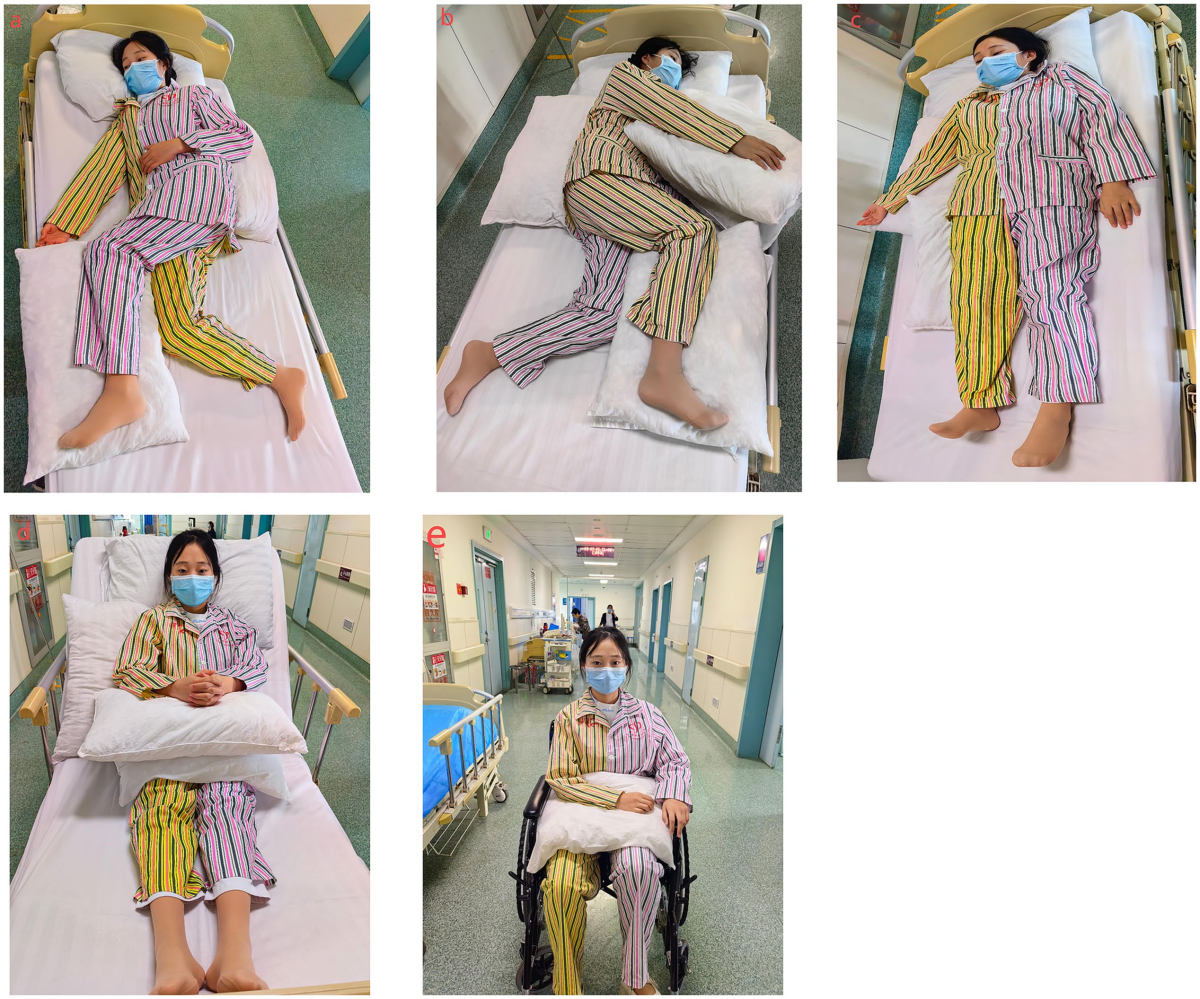
Five early rehabilitation positions for good limb positioning in hemiplegic patients, including side-lying, supine, bed sitting, and wheelchair sitting **(a–e)**. Yellow represents the hemiplegic side, while purple indicates the non-hemiplegic side.

Side-Lying Position with Non-Hemiplegic Limb Training: Patients are positioned in a side-lying posture with the hemiplegic side down and the non-hemiplegic side up. The shoulder on the hemiplegic side is protracted by 45° to prevent compression and ensure proper blood circulation. The hip and knee joints are slightly flexed to reduce the load on the hemiplegic limb. Non-hemiplegic limb training includes arm swings, wrist rotations, and finger flexion and extension exercises. Each activity consists of five repetitions per set, with ten sets performed daily to progressively enhance the function of the non-hemiplegic limb (Figure 1a). In order to prevent anterior-inferior shoulder subluxation—a common complication in stroke patients when the affected side is placed down—we additionally placed a specialized soft cushion under the hemiplegic shoulder during the side-lying position. This support helps to maintain proper shoulder alignment, reducing gravitational traction and thereby minimizing the risk of subluxation.

Side-Lying Position with Training for the Hemiplegic Limb: After completing non-hemiplegic limb training, patients are repositioned with the hemiplegic side up and the non-hemiplegic side down. A supportive pillow is placed at the waist to ensure comfort, with the legs flexed at 15° and a cushion positioned between the knees. Training for the arm, wrist, and fingers continues with the same regimen—five repetitions per set, performed ten times daily to maintain joint mobility and flexibility (Figure 1b).

Supine Position: In the supine position, thin pillows are placed under the hemiplegic shoulder and hip for support, and the head is slightly turned toward the hemiplegic side. The upper arm on the hemiplegic side is positioned in external rotation and abduction at 20°–40°, with the elbow and wrist extended, fingers outstretched, and palms facing upward. A pillow is placed beneath the knees, and the toes are directed upward to maintain alignment (Figure 1c).

Seated Position on the Bed: In the bed-seated position, the patient’s back, shoulders, arms, and lower limbs are supported by soft cushions, or the head of the bed is raised to 90°. The trunk should remain upright without forward leaning, with the elbows flexed at 90° and the hemiplegic arm extended forward. The knees are kept straight, and both arms are extended and rested on a meal tray or adjustable table (Figure 1d).

Wheelchair Seated Position: In the wheelchair-seated position, the patient sits with their back supported by the chair and the trunk upright, leaning slightly forward. The hemiplegic arm rests on a soft pillow placed in front of the chest, with the fingers naturally extended. To correct external rotation of the hemiplegic foot, the hip, knee, and ankle joints are positioned at 90° of flexion. The feet are flat on the floor, toes pointing forward, with both feet positioned shoulder-width apart (Figure 1e).

### Implementation of PDCA cycle

We initially attempted to implement Good Limb Positioning for all patients; however, this proved impractical in practice. Therefore, between July 1, 2022, and December 31, 2022, the PDCA cycle was introduced in the intervention group. A CQI team was established, comprising 12 members, including the Director of Rehabilitation Medicine, neurologists, rehabilitation physicians, therapists, and nurses. The PDCA cycle framework was applied to address barriers to the implementation of Good Limb Positioning in hemiplegic patients following thrombolysis for ACI.

#### Plan (P)

**Figure 2:**
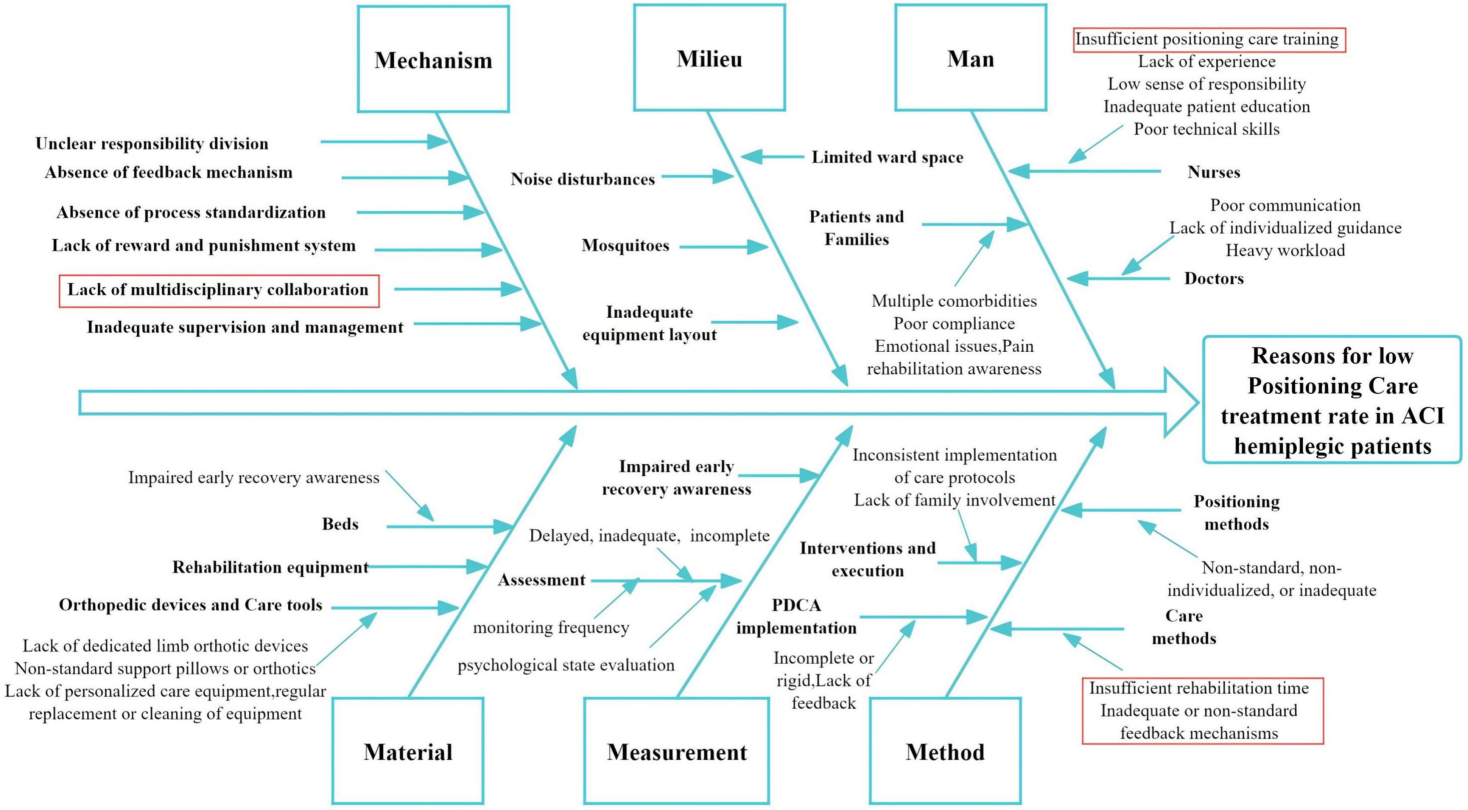
Fishbone analysis of reasons for low good limb positioning rate in ACI hemiplegic patients. Red highlights indicate the four primary causes.

A survey conducted on patients admitted during the first half of 2022 revealed that among 150 patients in the control group, only 72 (48%) received early rehabilitation therapy involving Good Limb Positioning, which fell significantly short of expectations. To identify the underlying causes of this low implementation rate, the CQI team, comprising neurologists, rehabilitation specialists, therapists, and nursing staff, conducted brainstorming sessions and on-site investigations. A root cause analysis (13) was performed using a fishbone diagram framework, focusing on six key dimensions: Mechanism (systematic workflows), Milieu (environmental conditions), Man (healthcare providers and patients), Measurement (evaluation protocols), Material (resources and equipment), and Method (standardization of care practices) (Figure 2). Prioritization of issues was performed using Pareto’s “80/20” principle, wherein each team member cast two votes to identify the most critical barriers. Four key factors emerged as priorities for intervention: lack of multidisciplinary collaboration, insufficient training in good limb positioning, inadequate allocation of rehabilitation time, and inconsistent or non-standard feedback mechanisms.

#### Development (D)

Targeted improvement strategies were subsequently developed and implemented after July 2022. These included specific interventions addressing each identified issue. Clinical staff applied the refined Good Limb Positioning protocols to optimize patient care. A detailed summary of strategies is provided (Table 1).

**Table 1:** Countermeasures for low good limb positioning rate in ACI hemiplegic patients.

Problems	Counter measures
Lack of multidisciplinary collaboration	1. Establish ACI rehabilitation teams with neurologists, rehabilitation physicians, therapists, and nurses, ensuring interdisciplinary collaboration. 2. Hold weekly joint consultations to discuss complex cases and optimize treatment strategies. 3. Organize workshops and seminars to promote interdisciplinary communication and align treatment goals. 4. Develop standardized protocols for multidisciplinary involvement in early rehabilitation. 5. Assign a designated coordinator to oversee and manage team collaboration and ensure timely communication. 6. Encourage information sharing among departments using digital platforms and regular meetings.
Insufficient training in good limb positioning	1. Provide hands-on workshops and simulation training for medical staff focusing on Good Limb Positioning techniques. 2. Create detailed instructional videos, manuals, and guidelines for standardized implementation. 3. Conduct periodic assessments of staff competency in positioning care, followed by refresher courses as needed. 4. Include patient and caregiver training in Good Limb Positioning to ensure continuity of care outside the hospital. 5. Use real-time monitoring and feedback tools to ensure correct application of limb positioning techniques. 6. Reward staff members who demonstrate excellence in positioning care practices to incentivize skill improvement.
Inadequate allocation of rehabilitation time	1. Optimize staff scheduling to ensure sufficient time allocation for each patient’s rehabilitation. 2. Develop individualized rehabilitation plans tailored to the severity of each patient’s condition. 3. Extend rehabilitation service hours and integrate therapy into routine nursing care to maximize time utilization. 4. Introduce time-tracking tools to monitor and evaluate the actual time spent on rehabilitation activities. 5. Implement real-time reminders and schedules for therapists and nurses to ensure adherence to planned rehabilitation activities.
Inconsistent or non-standard feedback mechanisms	1. Establish a standardized feedback framework to evaluate Good Limb Positioning practices and identify deficiencies. 2. Implement digital tracking tools to monitor patient progress, compliance, and rehabilitation outcomes. 3. Conduct regular audits and reviews to ensure adherence to standardized protocols. 4. Incorporate patient and caregiver feedback into care planning and process refinement. 5. Organize team discussions to share feedback and refine rehabilitation strategies.

#### Check (C)

The evaluation of the PDCA intervention was conducted by comparing key outcomes between the control and intervention groups, including patient adherence, self-efficacy, quality of life, patient satisfaction, and activities of daily living (ADL).

Self-efficacy was assessed using the General Self-Efficacy Scale (GSES), a validated 10-item instrument scored on a 4-point scale, with responses ranging from 1 (not at all true) to 4 (exactly true). Total scores were categorized as low (<20), moderate (20–30), and high (>30), with higher scores reflecting greater self-efficacy (14).

Quality of life was measured using the Medical Outcomes Study Short-Form 36 (SF-36), which evaluates functional health status across domains of physical, psychological, and social functioning. Scores range from 0 to 100, with higher scores indicating better quality of life (15).

Patient satisfaction was quantified using the Newcastle Satisfaction with Nursing Scale (NSNS), a 19-item survey scored on a 5-point Likert scale, yielding a total score between 19 and 95. Higher scores denote higher levels of satisfaction with nursing care (16).

ADL performance was evaluated using the Modified Barthel Index, which assesses independence in 10 daily activities, including feeding, bathing, dressing, bowel and bladder control, toileting, mobility, and stair climbing. Each activity is scored according to the level of independence, with a total possible score of 100; higher scores indicate greater functional independence (17).

Spasticity was evaluated using the Modified Ashworth Scale (MAS), a validated clinical tool for quantifying muscle tone in post-stroke patients (18). Trained assessors measured resistance to passive movement in key joints of the hemiplegic limb (including the shoulder, elbow, wrist, hip, knee, and ankle) at the time of discharge. MAS scores range from 0 (no increase in muscle tone) to 4 (severe increase with rigidity), and a score of 2 or above was defined as significant limb spasticity.

Outcome measures were collected at discharge to compare improvements in adherence rates, self-efficacy, quality of life, patient satisfaction, and ADL scores following the implementation of the PDCA-optimized Good Limb Positioning protocol. Adherence was defined as patients consistently following the prescribed limb positioning protocol and exercises throughout hospitalization, as monitored by nurses. Non-adherence was identified for patients who failed to comply with these requirements.

#### Assessment (A)

The outcomes were analyzed to determine the effectiveness of the implemented measures. Successful strategies were integrated into standardized clinical practices, while suboptimal interventions and newly identified challenges were addressed through iterative PDCA cycles for continuous improvement.

### Statistical analysis

Categorical variables were expressed as counts (n) and percentages (%), while continuous variables were presented as mean ± standard deviation (SD). Group differences were analyzed using the chi-square test for categorical data. Continuous data with a normal distribution were assessed using Student’s t-test, while non-normally distributed data were evaluated using the Mann–Whitney U test or Kruskal-Wallis test. Comprehensive statistical analyses were performed using SPSS software, version 26.0 (IBM Corporation, New York), and R software (version 4.3.3). A *p*-value of less than 0.05 was considered indicative of statistical significance.

## Results

### Baseline demographic and clinical characteristics

A total of 300 patients with hemiplegia following thrombolysis for acute cerebral infarction (ACI) were enrolled, including 150 in the control group and 150 in the study group. The mean age was 65.5 ± 12.2 years, with no significant difference between the control group (66.3 ± 10.5) and the study group (64.7 ± 13.7, *p* = 0.258). Post-thrombolysis NIHSS scores were similar between groups (15.3 ± 5.6 vs. 15.4 ± 5.4, *p* = 0.819). Hemoglobin levels (*p* = 0.447) and albumin levels (*p* = 0.309) showed no significant group differences. Gender distribution, comorbidities such as hypertension, diabetes, coronary heart disease, old cerebral infarction, atrial fibrillation, hyperlipidemia, and malignancies were comparable between the two groups (all *p* > 0.05) (Table 2).

**Table 2:** Baseline demographic of patients with hemiplegia following thrombolysis for ACI.

Variables	Overall (*n* = 300)	Control group (*n* = 150)	Study group (*n* = 150)	*p* value
Age, years	65.5 ± 12.2	66.3 ± 10.5	64.7 ± 13.7	0.258
Post-thrombolysis NIHSS, points	15.3 ± 5.5	15.3 ± 5.6	15.4 ± 5.4	0.819
Hemoglobin, g/L	140.3 ± 16.5	141.0 ± 17.5	139.6 ± 15.4	0.447
Albumin, g/L	40.9 ± 3.9	40.7 ± 4.1	41.2 ± 3.8	0.309
Gender				0.097
Female	116 (38.6%)	51	65	
Male	184 (61.4%)	99	85	
CHD				0.261
Yes	93 (31.0%)	51	42	
No	207 (69.0%)	99	108	
Diabetes				0.701
Yes No	85 (28.3%) 215 (71.7%)	44 106	41 109	
Hypertension				0.548
Yes No	191 (63.7%) 109 (36.3%)	98 52	93 57	
OCI				0.689
Yes No	75 (25.0%) 225 (75.0%)	36 114	39 111	
Atrial fibrillation				0.857
Yes No	35 (11.7%) 265 (88.3%)	17 133	18 132	
Malignant tumor				0.176
Yes No	5 (1.7%) 295 (98.3%)	4 146	1 149	
Hyperlipidemia				0.376
Yes No	22 (7.3%) 278 (92.7%)	9 141	13 137	

CHD, coronary heart disease; OCI, old cerebral infarction.

### Intervention outcomes

**Figure 3:**
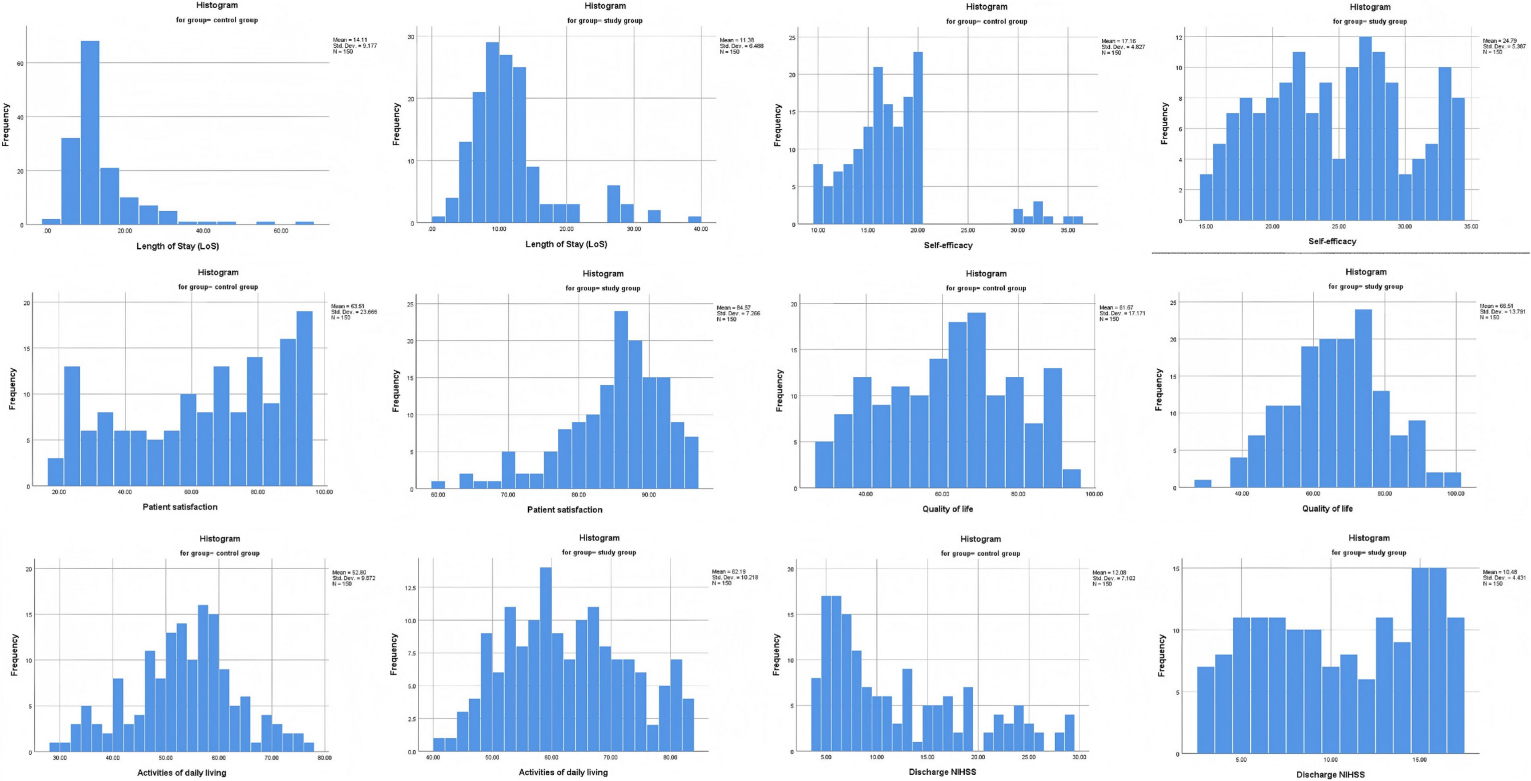
Histograms of Intervention Outcomes and Prognostic Data Normality Assessment. The normality of prognostic data was assessed using the Kolmogorov–Smirnov (K-S) test, with histograms illustrating the distribution. Quality of life and activities of daily living data demonstrated good normality (*p* > 0.05).

Patient adherence to Good Limb Positioning on Early Functional Recovery significantly improved following the implementation of the PDCA-optimized protocol. Specifically, in the control group, the adherence rate was 48.0% (72/150), whereas the study group achieved 88.0% (132/150) (*χ*^2^ = 55.1, *p* < 0.001). Prior to analysis, normality testing was conducted, revealing that Quality of Life and Activities of Daily Living followed a normal distribution, whereas Self-efficacy and Patient Satisfaction did not (Figure 3). Moreover, patient satisfaction scores demonstrated a notable increase in the study group following the implementation of the PDCA-optimized protocol. For instance, the median satisfaction score was 86.0 (81.0, 90.0) in the study group, compared to 68.5 (43.5, 84.3) in the control group (*Z* = −7.812, *p* < 0.001), reflecting enhanced perceptions of care quality and improved patient-provider interactions after PDCA implementation. In addition, self-efficacy scores, reflecting patients’ confidence in managing their condition, were significantly higher in the study group, with a median score of 25.0 (20.0, 29.0) compared to 17.0 (14.0, 19.0) in the control group (*Z* = −11.013, *p* < 0.001). This indicates that targeted care strategies under the PDCA framework effectively enhanced patient autonomy and engagement in rehabilitation. Furthermore, quality of life, assessed using the SF-36 scale, also showed substantial improvement. The study group achieved a mean score of 66.5 ± 13.8, compared to 61.7 ± 17.2 in the control group (*t* = −2.692, *p* < 0.001), highlighting better outcomes in physical, psychological, and social functioning following the structured intervention. Lastly, functional independence, measured by the ADL score, demonstrated marked improvements in the study group. Specifically, the mean ADL score was 62.2 ± 10.2, which was significantly higher than the control group’s 52.8 ± 9.9 (*t* = −8.097, *p* = 0.008). This reflects enhanced capability to perform daily activities and indicates better overall recovery (Table 3).

**Table 3:** Comparison of intervention outcomes between two groups of patients.

Variable	Overall (*n* = 300)	Control group (*n* = 150)	Study group (*n* = 150)	χ^2^/t/Z	*p*-value
Patient adherence				55.1	0.000
Yes	204	72	132		
No	96	78	18		
Patient satisfaction, points 82.0 (66.3,88.0)		68.5 (43.5, 84.3)	86.0 (81.0, 90.0)	−7.812	0.000
Self-efficacy, points 20.0 (16.0,26.0)		17.0 (14.0, 19.0)	25.0 (20.0, 29.0)	−11.013	0.000
Quality of life, points 64.0 ± 15.7		61.7 ± 17.2	66.5 ± 13.8	−2.692	0.000
Activities of daily living, points 57.4 ± 11.0		52.8 ± 9.9	62.2 ± 10.2	−8.097	0.008

### Comparison of the prognosis of the two groups

**Figure 4:**
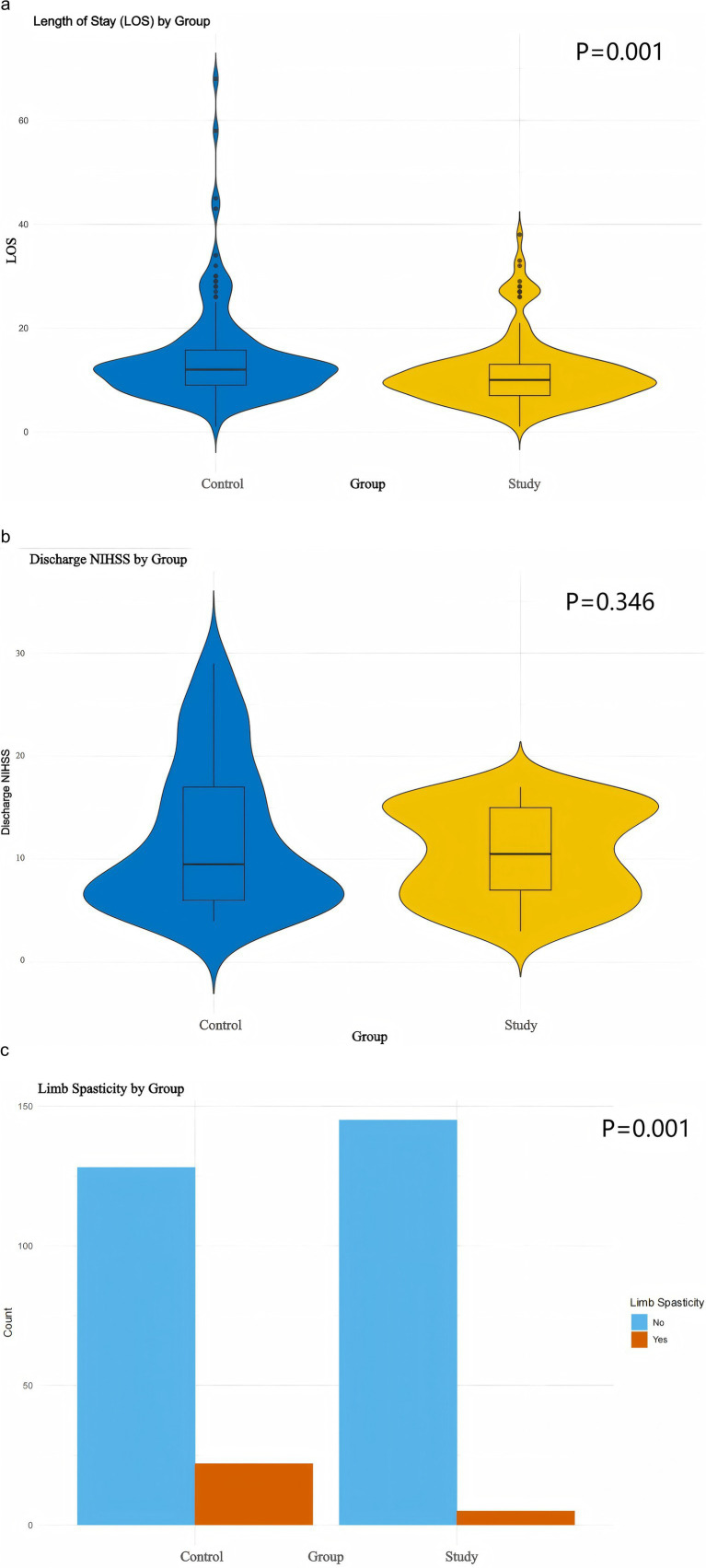
Prognostic visualization. **(a,b)** Violin plots with Mann–Whitney U test. **(c)** Box plots with Chi-square test.

The outcomes of Good Limb Positioning implementation under the PDCA framework were further assessed by analyzing discharge NIHSS scores, length of stay (LoS), and the occurrence of limb spasticity in patients with acute cerebral infarction (ACI) following thrombolysis. Both discharge NIHSS scores and LoS were found to deviate from normal distribution (Figure 3) and were analyzed using the Mann–Whitney U test. The median LOS was significantly reduced in the study group (10, 7–13 days) vs. (12, 9–12 days) in the control group (*Z* = −3.321, *p* = 0.001) (Figure 4a). However, no significant difference was observed in discharge NIHSS scores between the study group (9.5, 6–17) vs. (10.5, 6.8–15) in the control group (*Z* = −0.942, *p* = 0.346) (Figure 4b). In contrast, the incidence of significant limb spasticity demonstrated a statistically significant reduction in the study group compared to the control group (*χ*^2^ = 11.762, *p* = 0.001) (Figure 4c).

## Discussion

ACI, a leading cause of disability and mortality in elderly populations, imposes substantial physical and psychological burdens on patients due to the high prevalence of hemiplegia and associated motor dysfunctions following stroke. Stroke survivors often experience persistent impairments in motor pathways, significantly reducing their quality of life and necessitating comprehensive rehabilitation interventions (7). Early rehabilitation, particularly through good limb positioning, plays a critical role in mitigating complications such as shoulder-hand syndrome, muscle contractures, and foot drop, while simultaneously supporting neural plasticity and motor recovery (19). Studies have demonstrated that systematic and early interventions, including proper limb alignment and positioning, not only prevent secondary complications but also accelerate functional improvements by enhancing neuronal excitability and promoting the recovery of motor pathways (3, 19). Furthermore, the integration of tailored rehabilitation protocols, such as those guided by the PDCA framework, has proven to enhance adherence and ensure the implementation of standardized, effective practices (8). Good limb positioning is a particularly effective strategy within early rehabilitation, offering benefits such as improved physiological function, prevention of joint and soft tissue complications, and enhanced overall recovery trajectories for patients with ACI (20). Given the profound impact of ACI on individual and societal levels, emphasizing the necessity of early rehabilitation measures, particularly good limb positioning, is paramount to improving clinical outcomes and reducing the long-term burden of stroke.

In stroke rehabilitation, clinical practice guidelines (CPGs) often recommend early mobilization, but the specifics of implementation can vary widely across healthcare settings. Local practices may influence how early mobilization interventions are applied, and this variability can potentially impact the effectiveness of the intervention (21). The timing, frequency, and type of mobilization recommended by different institutions can vary, resulting in different outcomes for patients. For instance, some hospitals may begin mobilization within the first 24 h post-stroke, while others may wait until the patient’s condition stabilizes, which could lead to variations in recovery rates. Additionally, factors such as staff training, the availability of rehabilitation resources, and the level of multidisciplinary collaboration play a significant role in the success of early mobilization practices. Hospitals with well-coordinated teams, including physiatrists, neurologists, nurses, and physiotherapists, may have more structured and consistent implementation of early mobilization protocols compared to hospitals where such coordination is lacking. Research has shown that hospitals with high levels of multidisciplinary collaboration tend to see better functional recovery in stroke patients, as coordinated care ensures that each aspect of the patient’s rehabilitation is addressed comprehensively (22).

Another challenge in the application of early mobilization is the patient’s individual condition. Stroke patients are highly heterogeneous in terms of their severity, comorbidities, and response to rehabilitation, which requires a more personalized approach to mobilization. Guidelines that are flexible enough to allow for customization based on the patient’s clinical status are more likely to lead to better outcomes. Conversely, overly rigid protocols may not be suitable for all patients and could lead to suboptimal recovery. In light of these issues, standardizing early mobilization protocols while allowing for individualized adaptations based on patient needs could help mitigate variability across healthcare settings. Ensuring that healthcare workers are trained in the latest evidence-based mobilization techniques and emphasizing the importance of early, coordinated intervention may enhance the effectiveness of stroke rehabilitation and improve recovery outcomes on a broader scale.

The PDCA cycle quality management method has been widely applied in clinical practice, promoting the transition from evidence-based to more systematic and scientific nursing care while improving overall care quality. For instance, its integration into operating room practices has effectively reduced incision infection rates and irregular events, while enhancing disinfection standards and nursing quality (23). In digestive endoscopy nursing, PDCA implementation has lowered infection rates, improved disinfection protocols, increased patient satisfaction, and strengthened both nursing safety and operational skills (24). Similarly, in hemodialysis care, the PDCA cycle has been shown to improve patient satisfaction and significantly reduce vascular-related complications, including internal fistula obstruction, thrombosis, and infection (25). Furthermore, its application in daytime varicocelectomy has shortened hospitalization time, reduced costs, and improved patient satisfaction, demonstrating its value in optimizing time-sensitive surgical procedures (26).

The results of this study showed that after implementing the PDCA cycle management model for Good Limb Positioning, the observation group exhibited significantly higher self-efficacy and ADL scores compared to the control group (*p* < 0.05). The underlying reasons include the PDCA cycle’s emphasis on setting higher standards and requirements for nurses, ensuring not only professional competence but also proficiency in operational skills. Additionally, during the care process, the model facilitates timely identification of issues and deficiencies, with corresponding corrective measures proposed to minimize the risk of adverse events, safeguard patient safety, and accelerate recovery. The study also observed a reduction in the median LoS in the observation group, decreasing to 10 days compared to 12 days in the control group. This reduction indicates that the structured protocol not only enhances functional recovery but also shortens rehabilitation timelines, potentially reducing healthcare costs and resource consumption. This is consistent with the findings of previous studies (27, 28). Although there was no significant difference in discharge NIHSS scores between the two groups, the incidence of limb spasticity was significantly lower in the observation group, demonstrating the PDCA model’s effectiveness in preventing secondary complications in ACI patients. As a modern and scientifically driven management approach, the PDCA cycle adopts an iterative, step-by-step improvement strategy that aligns well with standardized nursing workflows. When applied to the clinical care of stroke patients with hemiplegia, the PDCA model not only provides targeted nursing interventions and accurate guidance for rehabilitation and limb positioning but also significantly contributes to improving patients’ physical condition, restoring motor function, and creating better conditions for their reintegration into society and independent living (29).

To improve the implementation of good limb positioning in stroke care, several practical strategies can be employed. First, nursing staff must be both organizers and implementers of the process. This dual role requires not only mastering relevant knowledge and technical skills but also enhancing communication and education capabilities. Nurses should actively educate patients and their families about the significance and necessity of proper limb positioning to encourage adherence and cooperation. Additionally, they must identify factors that impact the effectiveness of limb positioning and adjust interventions continuously to achieve optimal outcomes. The integration of technology holds significant potential for improving communication and compliance. For instance, leveraging internet-based platforms, educational videos, and mobile applications such as WeChat can facilitate more accessible and effective interactions between healthcare providers and patients. Interactive tools like digital whiteboards can also enhance understanding and engagement during rehabilitation education (30). Moreover, incorporating advanced rehabilitation technologies, such as exoskeleton rehabilitation robots designed for positioning and movement training, could significantly enhance the precision and efficacy of care by improving balance and motor function (31).

Several limitations should be acknowledged. First, individual patient characteristics, such as stroke subtype, severity, and comorbidities, which may significantly impact rehabilitation outcomes, were not fully stratified in this study. Additionally, the study did not account for other interventions patients may have received during their hospital stay, such as standard care or similar rehabilitation methods. Future research could consider personalized rehabilitation plans based on these factors. Despite using standardized assessment tools (e.g., GSES, SF-36), subjective evaluations like patient satisfaction and adherence may still be influenced by bias, potentially overestimating or underestimating the effects of good limb positioning. Future studies should incorporate more objective measures, such as clinical functional assessments and biomarkers, to validate the intervention’s impact. Moreover, no follow-up or post-discharge surveillance was performed to assess potential residual or carry-over effects. Additionally, the relatively small sample size and regional nature of this study, along with its observational design, introduce inherent limitations and biases. Further multicenter and multidisciplinary research is needed to deepen our understanding of good limb positioning care and its broader applicability in clinical settings.

## Conclusion

This study suggests that the PDCA cycle management model may enhance Good Limb Positioning in early rehabilitation for hemiplegic patients with acute cerebral infarction. By addressing adherence barriers, the PDCA approach may modestly improve functional recovery, reduce secondary complications, and shorten rehabilitation time, highlighting the potential role of quality improvement frameworks in clinical protocols. Future research should explore the PDCA model’s broader application in healthcare and its effects on long-term recovery and quality of life in stroke patients.

## Data Availability

The original contributions presented in the study are included in the article/supplementary material, further inquiries can be directed to the corresponding author/s.
